# Supplementary material for: Stacking Model‐Based Classifiers for Dealing With Multiple Sets of Noisy Labels
Source: Biom J. 2025 Mar 12;67(2):e70042. doi: 10.1002/bimj.70042 (PMC11898607; doi:10.1002/bimj.70042)
Supplement: Supplementary file 1 — Supporting Information [file BIMJ-67-e70042-s001.zip › density-based-ensemble-model-main/competitors_real_data/netlab-master/netlab-master/help/som.htm]

Netlab Reference Manual som


# som

## Purpose

Creates a Self-Organising Map.

## Synopsis

```
net = som(nin, map_size)
```

## Description

`net = som(nin, map_size)` creates a SOM `net`
with input dimension (i.e. data dimension) `nin` and map dimensions
`map_size`. Only two-dimensional maps are currently implemented.

The fields in `net` are

```
  type = 'som'
  nin = number of inputs
  map_dim = dimension of map (constrained to be 2)
  map_size = grid size: number of nodes in each dimension
  num_nodes = number of nodes: the product of values in map_size
  map = map_dim+1 dimensional array containing nodes
  inode_dist = map of inter-node distances using Manhatten metric
```

The map contains the node vectors arranged column-wise in the first
dimension of the array.

## See Also

`kmeans`, `somfwd`, `somtrain`

---

**Pages:**
Index


---

Copyright (c) Ian T Nabney (1996-9)
